# Image‐guided helical tomotherapy for localized prostate cancer: technique and initial clinical observations

**DOI:** 10.1120/jacmp.v8i3.2320

**Published:** 2007-07-17

**Authors:** Chester R. Ramsey, Daniel Scaperoth, Rebecca Seibert, Daniel Chase, Thomas Byrne, Stephen Mahan

**Affiliations:** ^1^ Thompson Cancer Survival Center Knoxville Tennessee U.S.A.; ^2^ The University of Tennessee Knoxville Tennessee U.S.A.

**Keywords:** Prostate, image‐guided radiation therapy, helical tomotherapy, intensity modulation

## Abstract

The purpose of the present study was to implement a technique for daily computed tomography (CT)–based image‐guided radiation therapy and to report observations on treatment planning, imaging, and delivery based on the first 2 years of clinical experience.

Patients with previously untreated stage T1 – T3 biopsy‐proven adenocarcinoma of the prostate were considered eligible for treatment with daily CT‐guided helical tomotherapy. The prostate was targeted daily using megavoltage CT (MVCT) images that were fused with treatment‐planning CT images based on anatomic alignments. All patients were treated at 2 Gy per fraction to 76 – 78 Gy (mean: 76.7 Gy).

As part of this study, 33 prostate patients were planned, imaged, and treated with a total of 1266 CT‐guided fractions. The prostate, rectum, bladder, femoral heads, and pubis symphysis were visible in one or more slices for all 1266 MVCT image sets. The typical range of measured prostate displacement relative to a 3‐point external laser setup in this study was 2 – 10 mm [3.4 mm standard deviation (SD)] in the anterior–posterior direction, 2 – 8 mm (3.7 mm SD) in the lateral direction, and 1 – 6 mm (2.4 mm SD) in the superior–inferior direction. The obese patients in this study had a substantially larger lateral variation (8.2 mm SD) attributable to mobility of skin marks. The prostate, seminal vesicles, rectum, and bladder anatomy were used to position the patient relative to the desired treatment position without the use of implanted markers. Acute toxicities were within the expected range given the number of patients treated and the dose level.

PACS numbers: 87.50.Gi, 87.53.Mr, 87.53.Tf

## I. INTRODUCTION

Recent advances in computed tomography (CT), magnetic resonance imaging (MRI), and ultrasound‐based image‐guided radiation therapy (IGRT) have improved the understanding of the dynamic nature of prostate, bladder, and rectum position during the course of radiotherapy.^(1– 24)^ The three‐dimensional (3D) conformal radiation therapy and intensity‐modulated radiation therapy (IMRT) treatment techniques require margins to compensate for internal prostate motion and setup error. Unfortunately, larger treatment margins result in a greater volume of bladder and rectum receiving doses in excess of 70 Gy, and the limiting factor in dose escalation for the prostate is the increased occurrence of unacceptable toxicities with increasing dose.

The development and use of daily pretreatment IGRT has increased dramatically over the past 5 years because of a desire to compensate for prostate positional uncertainty. Systems for IGRT use ultrasound, CT, cone‐beam CT, and orthogonal planar X‐ray imaging to localize the prostate daily before treatment delivery.^(^ ^1^ ^–^ ^24^ ^)^ One such system is the HI‐ART^2^ Helical Tomotherapy (TomoTherapy, Madison, WI) delivery system.^(^ ^25^ ^–^ ^29^ ^)^

Helical tomotherapy is a new rotational radiotherapy delivery system that has the ability to obtain CT images of the patient in the treatment position before each treatment.^(^ ^20^ ^,^ ^30^ ^,^ ^31^ ^)^ Like other IGRT systems, tomotherapy can use pretreatment imaging to correct for the positional uncertainties associated with setup error and interfraction organ motion. The position of the target volume relative to the treatment isocenter can then be corrected by moving the patient with appropriate offsets.

Systems for IGRT have the potential to allow for a reduction in the planned target volume, which may significantly reduce dose to adjacent normal structures. And an additional advantage of CT‐based IGRT systems (such as tomotherapy and cone‐beam CT) is that the CT images can be used for dose recalculation of each treatment fraction.^(32)^ Dose recalculation allows for daily variation in the delivered doses to the target volume and normal tissues to be assessed.

The present study reports on the first 2 years of clinical experience using daily CT‐guided helical tomotherapy for prostate radiotherapy. Specifically, the planning technique, imaging technique, treatment technique, and initial clinical observations are reported.

## II. PATIENTS AND METHODS

Patients with previously untreated, biopsy‐proven adenocarcinoma of the prostate were imaged and treated with CT‐based image‐guided helical tomotherapy. Patients with all 1992 American Joint Committee on Cancer clinical stages T1 – T3 cancers were eligible for inclusion, except for those who were felt to have such a favorable prognosis with conventional radiation therapy that higher doses were not justified.^(^ ^33^ ^,^ ^34^ ^)^ Neoadjuvant and adjuvant hormone therapies were allowed. However, neoadjuvant hormone therapy had to be initiated 2 – 6 months before initiation of radiotherapy treatment.

### A. Treatment Planning

Treatment‐planning CT images were acquired on a kilovoltage CT simulator (General Electric, Milwaukee, WI) with the patient positioned supine and immobilized with a knee sponge and foot holder. Additional immobilization was felt to be unnecessary, because daily CT imaging was to be used to position the patient for treatment. Treatment‐planning CT images were acquired at or above the level of the iliac crest inferior through the perineum. All tissue to be irradiated was included in the CT scan. The CT scan thickness was required to be 0.5 cm or less through the region that contained the target volume. With few exceptions, simulation occurred within 1 week of the initiation of radiation therapy.

The gross tumor volume (GTV) was defined as the entire prostate gland as visualized on the CT scan. The seminal vesicles were included in the GTV for the first 50 Gy of the prescription dose if the estimated risk for seminal vesicle involvement was greater than 10%.^(35)^ The clinical target volume (CTV) was considered to be the same as the GTV for patients with a less‐than 15% risk of nodal involvement. The risk of nodal involvement was estimated using this relationship developed by Roach et al.^(36)^:

Risk of nodel involvement=(2/3)PSA+[(GS−6)×10] ,

where PSA is the patient's prostate‐specific antigen level (ng/mL) and GS is the patient's Gleason score.

For patients with a greater than 15% risk of nodal involvement, the CTV included the pelvic lymph nodes.^(37)^ The nodal CTV was defined by adding a 2‐cm margin around the iliac arteries from L5 to the inferior aspect of the pelvis. The planning target volume (PTV) for all patients was defined by adding 5‐mm margin around the CTV posterior, and a 10‐mm margin in all other directions. These margins are often used as standard margins for modern 3D conformal radiation therapy.^(33)^ Conventional margins were used for the first group of prostate patients treated, because the adequacy of the MVCT imaging was unknown when this study was initiated in August 2003.

All inverse treatment planning was performed using the TomoTherapy HI‐ART^2^ treatment planning system, which consists of a parallel‐processing computer system with 32 processors. The primary jaw setting was 2.5 cm, the pitch setting was 0.333, the modulation factor was 2.5, and the calculation resolution was set to “normal” (2‐ to 4‐mm voxels). The prescription dose was defined as 2.0 Gy per fraction to 90% of the PTV to 50 Gy for the initial portion of the treatment, and then 26 – 28 Gy additional dose to 90% of the boost PTV. The minimum dose to the PTV was no less than 95% of the prescribed dose, and the minimum dose to the CTV was no less than 2.0 Gy per fraction. No more than 2% of the PTV was to exceed the prescribed dose by 7%.

Contoured normal‐tissue volumes included the bladder, rectum, femoral heads, and 2 cm of miscellaneous normal tissue above and below the PTV. Patients were instructed to have a full bladder and an empty rectum before CT simulation and treatment delivery. The bladder was contoured from apex to dome, and the rectum was contoured from the anus (at the level of the ischial tuberosities) for a length of 15 cm, or to the point at which the rectosigmoid flexure could be identified. All normal tissues were considered solid organs.

The volume of the critical structures intersected by the PTV were considered in the dose– volume histograms (DVHs) of target and normal tissues alike. In tomotherapy delivery without a defined border, the superior–inferior dose gradients tend to be broad. The miscellaneous normal‐tissue structure was therefore used to maximize the superior–inferior dose gradient. This normal structure was also used in maximizing the conformal avoidance of the bladder and the penile bulb.

The tomotherapy inverse planning system uses an interactive optimization interface that takes 3 – 6 seconds per iteration after completion of the beamlet pre‐calculation. The optimization goals for the rectum were no more than 40% receiving more than 46 Gy,^(38)^ and no more than 15 cm^3^ of rectum receiving more than 70 Gy.^(39)^ The optimization goals for the bladder were no more than 30% of the bladder receiving more than 65 Gy.^(33)^ The treatment planner interactively adjusted the critical structure objectives to minimize doses to the normal tissues without exceeding the foregoing CTV and PTV dose‐uniformity constraints.

### B. Image guidance

**Figure 1:**
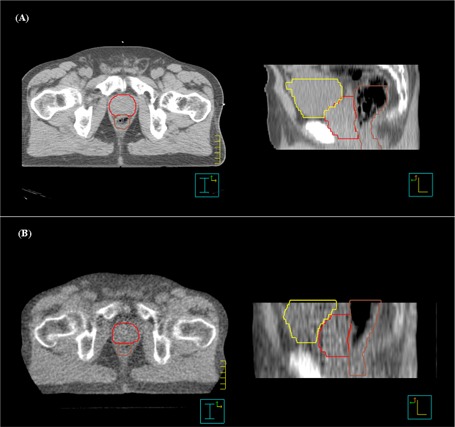
Comparison of (A) diagnostic computed tomography (CT) and (B) megavoltage CT images taken on the HI‐ART^2^ system (TomoTherapy, Madison, WI) for axial (left panel) and sagittal (right panel) views. The prostate, rectum, and bladder contours are shown. Note the superior–inferior displacement of the prostate relative to the reference treatment‐planning CT.

Patients were placed in the treatment position on the treatment table by aligning external skin marks with wall‐mounted red lasers. The MVCT images were acquired from at least 1 cm below the CTV to 1 cm above the CTV using either a 2.5‐mm or 4.0‐mm slice thickness. Fig. 1 shows an example of the MVCT image quality for the prostate in the axial and sagittal planes. After the MVCT images had been reconstructed, they were automatically fused with the treatment‐planning CT images on the operator station using a full‐image pixel‐by‐pixel analysis to co‐register the images.^(40)^ The translations were calculated using the full‐image technique with the “standard” resolution setting on the operator station.

**Figure 2:**
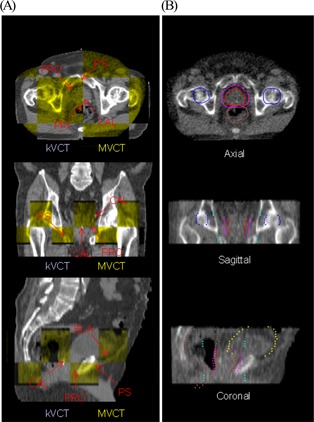
The image fusion tool on the TomoTherapy (Madison, WI) operation station is used to register the treatment planning kilovoltage computed tomography (kVCT) images with megavoltage computed tomography (MVCT) images. After an automated CT‐to‐CT fusion, the operator can manually adjust the registration (A) by using a “checkerboard” display or (B) by overlaying the treatment planning contours with the MVCT image. Axial, sagittal, and coronal views are shown for both registration techniques. Arrows in the “checkerboard” display point to anatomic landmarks used in manual adjustments for prostate patients. The prostate (red), planning target volume (pink), rectum (brown), bladder (yellow), and femoral heads (blue) are shown in the contour overlay. PRO=prostate; PS=pubis symphysis; CAL=calcification; PRI=prostate−rectal; BLA=bladder.

The image fusion was reviewed before treatment, and manual adjustments were made if necessary. Manual adjustments were often necessary for prostate IGRT because the automatic tomotherapy fusion algorithm uses the full CT image and is not specifically targeting the prostate and rectum. The manual alignment was performed by using a fusion split screen display to visually inspect the prostate, seminal vesicles, rectum, and bladder (Fig. 2). The reviewer scrolled through multiple axial, sagittal, and coronal CT slices to ensure that the prostate and seminal vesicles were correctly aligned. In a departure from previous tomotherapy imaging studies, implant markers were not used in this study.^(20)^

After the completion of image fusion, the patient was automatically repositioned for treatment delivery by the tomotherapy system based on the calculated anterior–posterior and superior–inferior shifts. The radiation therapists manually moved the treatment couch in the right–left lateral direction. All patient repositioning was performed by moving the couch, not the patient. Roll corrections were implemented for approximately half the patients in the study once software was released for adjusting the gantry start‐angle positions. For these patients, the automatic image fusion was performed using the translations plus roll option.

After couch repositioning, the patients in this study were not rescanned to confirm correct application of the shifts. Rescans were not performed for these reasons:

- Other imaging studies at our institution that used repeat scanning have shown that the shifts are consistently applied correctly.^(30)^
- Conventional margins were used in the present study.
- Increasing the imaging time would increase the probability of intrafraction motion.^(^ ^41^ ^,^ ^42^ ^)^

### C. Treatment delivery

Treatment was delivered using a helical delivery sequence. Delivery of IMRT used a 2.5×40.0‐cm rotating fan beam generated by a 6‐MV linear accelerator, a 64‐leaf air‐actuated binary multileaf collimator, and an image detector array mounted on a rotating slip‐ring. Treatment delivery was accomplished by rotating the slip‐ring at a constant angular velocity while the couch moved into the bore with a pitch of 0.333. Modulation was achieved by varying the length of time that each leaf spends in and out of the treatment beam during gantry rotation for 51 discrete projections per rotation.

Side effects were assessed weekly during treatment and at follow‐up by the same radiation oncologist (DSS). Effects occurring within 120 days from the start of therapy were considered to be acute radiation morbidity. These acute radiation‐induced toxicities were scored according to a modified Radiation Therapy Oncology Group (RTOG) acute radiation morbidity scoring criteria (Table 1).^(43)^

**Table 1:** Modified Radiation Therapy Oncology Group grading criteria

	Acute genitourinary	Acute gastrointestinal
Grade 1	Requiring no medication	Requiring no medication
Grade 2	Frequency of urination or nocturia less frequent than every hour, dysuria, urgency, bladder spasm requiring local anesthetic	Diarrhea requiring parasympatholytic drugs; mucous discharge not necessitating sanitary pads; rectal or abdominal pain requiring narcotic analgesics
Grade 3	Frequency with urgency and nocturia hourly or more frequently, dysuria, pelvis pain, or bladder spasm requiring frequent narcotic; gross hematuria with or without clot formation	Diarrhea requiring parenteral support; severe mucous or blood discharge necessitating sanitary pads; abdominal distension
Grade 4	Hematuria requiring transfusion; acute bladder obstruction not secondary to clot passage, ulceration, or necrosis	Acutre or subacute obstruction, fistula or perforation; gastrointestinal bleeding requiring transfusion; abdominal pain or tenesmus requiring tube decompression or bowel diversion

## III. RESULTS AND DISCUSSION

### A. Treatment planning

A full course of helical tomotherapy with at least 1 year of follow‐up was used to definitively treat 33 prostate patients (Table 2). Of these 33 patients, 30 were initially treated with a CTV that consisted of the prostate and seminal vesicles to a prescribed dose of 50 Gy. The 3 patients that were at risk for nodal involvement were initially treated to 50 Gy using a tomotherapy‐based IMRT pelvis treatment. All 33 patients were treated with a 26 – 28 Gy boost where the PTV consisted of the prostate CTV plus margins.

**Table 2:** Patient characteristics (n=33)

Characteristic	*n* (%)
Age (years)	
<70	8 (24)
≥70	25 (76)
T‐Stage	
I	28 (85)
II	4 (12)
III	1 (3)
Gleason score	
2–5	1 (3)
6–7	31 (96)
8–10	1 (3)
Initial PSA (ng/mL)	
<10	27 (82)
10–20	6 (18)
Hormones	
Yes	24 (73)
No	9 (27)
Follow‐up (months)	
Minimum	14.3
Mean	24.0
Maximum	38.5

**Figure 3:**
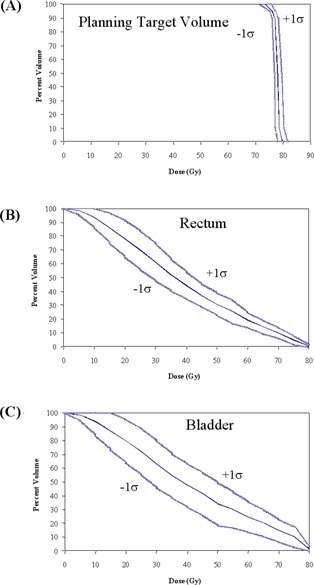
Dose–volume histograms (solid blue lines) and the standard deviation (light blue lines) for (A) mean planning target volume (PTV), (B) rectum, and (C) bladder for 30 prostate tomotherapy patients. The PTV consists of the prostate with a 5‐mm posterior margin and a 10‐mm margin in all other directions.

Fig. 3 shows the mean PTV, rectum, and bladder DVHs for the 30 patients whose initial CTV consisted of the prostate and seminal vesicles. In addition to the mean DVHs, the standard deviation for each structure is also shown. For tomotherapy delivery, the mean volume of rectum receiving more than 70 Gy was 10%, and the mean volume of bladder receiving more than 65 Gy was 20%. Mahan et al. recently measured the dose gradient of a helical tomotherapy system and found a gradient of 10% per millimeter with a PTV uniformity index of less than 11%.^(30)^ The helical rotational nature of this form of IMRT delivery allows the beamlets to be directed at the target from 51 projections per gantry rotation. The result is increased conformal avoidance without a reduction in dose uniformity inside the PTV.

**Figure 4:**
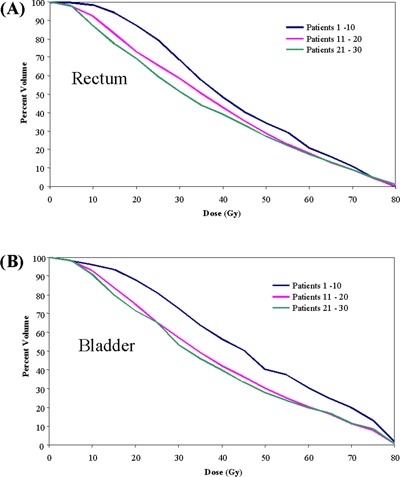
Dose–volume histograms of mean (A) rectum and (B) bladder for 30 prostate tomotherapy patients sorted chronologically into groups of ten. The level of rectal and bladder conformal avoidance increased with inverse treatment planning experience.

To determine the impact of experience in the treatment planning process, patients in this study were sorted chronologically. Fig. 4 shows the mean rectum and bladder DVHs as a function of when the patients were treated. Beyond the first 10 patients, conformal avoidance of the bladder substantially improved. Initially, the optimization constraints used during tomotherapy inverse planning were the same as those that had been used for fixed‐gantry IMRT. For example, no more than 30% of the bladder was to receive more than 65 Gy.^(33)^ The tomotherapy conformal avoidance was increased by using more aggressive optimization parameters. By changing the bladder constraints, the volume of bladder receiving more than 60 Gy was decreased to 20% from 31%. In the low‐dose region, the volume of bladder receiving more than 40 Gy was reduced to 40% from 57%. The rectal DVHs also improved with tighter objectives. The volume of rectum receiving more than 65 Gy was reduced to 13% from 16%, and the volume of rectum receiving more than 30 Gy was reduced to 52% from 69%. This additional conformal avoidance of the bladder and rectum was achieved without compromising PTV coverage or dose uniformity.

**Figure 5:**
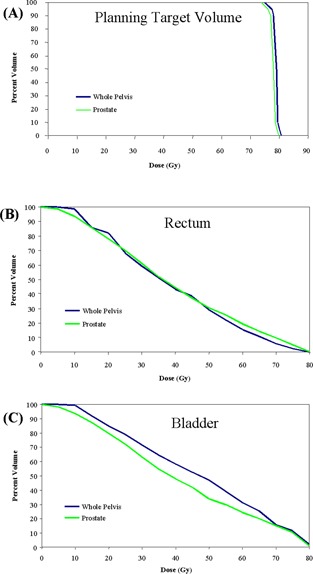
Dose–volume histograms of mean (A) planning target volume (PTV), (B) rectum, and (C) bladder for 30 prostate tomotherapy patients with the initial clinical target volume (CTV) comprising the prostate and seminal vesicle and for 3 tomotherapy patients whose initial CTV included at‐risk pelvic lymph nodes.

Three of 30 patients were treated with a tomotherapy‐based whole‐pelvis technique to 50 Gy. Fig. 5 shows a comparison of the mean rectal and bladder DVHs for the patients treated initially with the tomotherapy whole‐pelvis field and the patients treated initially to the prostate and seminal vesicles only. Because of the high‐dose gradients that are achievable with helical tomotherapy, the mean rectal DVHs are essentially equivalent for the two delivery techniques. As expected, the bladder DVHs were greater for the tomotherapy‐based whole‐pelvis technique. Because the superior border of the treatment field extended to L5, the PTV surrounded the entire bladder and a greater amount of the bladder was included in the PTV. Further analysis of the tomotherapy‐based whole‐pelvis technique will be needed because of the small sample size in the present study.

### B. Image guidance

The beam‐on time required to acquire a single CT slice on the tomotherapy system was 5 s. The tomotherapy system has three image acquisition modes (“fine,” “normal,” and “coarse”) that correspond to slice thicknesses of 2.5 mm, 4.0 mm, and 6.0 mm. The imaging beam‐on time can be estimated by dividing the desired superior–inferior imaging distance by the slice thickness and then multiplying the resulting number of desired slices by 5 s per slice. All patients in the present study were imaged with 2.5‐mm or 4.0‐mm slices and had imaging beam‐on times ranging from 2 to 3 minutes. The additional time for image reconstruction, automatic registration, manual review, and patient setup corrections ranged from 3 to 5 minutes. The total time between the start of imaging and the beginning of treatment delivery was therefore a mean of 7.1 minutes (range: 6.2 – 8.5 minutes).

**Figure 6:**
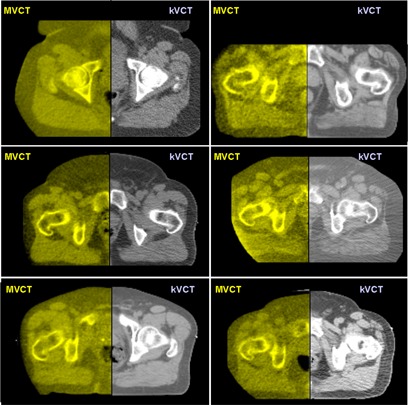
Six random examples of axial megavoltage computed tomography (MVCT)–to–kilovoltage computed tomography (kVCT) registration for prostate patients using the “checkerboard” image fusion tool on the TomoTherapy (Madison, WI) operator station. The right half of each prostate image is the reference kVCT (grayscale), and the left half is the daily MVCT image (yellowscale). Note that the prostatic–rectal interface is visible in each image.

The prostate, rectum, bladder, femoral heads, and pubis symphysis were visible in one or more slices for all 1266 MVCT image sets. Fig. 6 shows a random sampling of axial MVCT‐to‐kilovoltage CT (kVCT) registrations for the prostate patients treated in this study. A “checkerboard” image fusion tool on the tomotherapy delivery console was used to perform the manual alignment before treatment delivery. The right half of each prostate image fusion is the reference kVCT (grayscale), and the left half is the daily MVCT image (yellowscale). Intrafraction motion attributable to respiration and bowel motion did not produce artifacts that rendered the images unusable. Because the MVCT images are acquired at a rate of one slice every 5 s, when motion artifacts were observed, they were limited to one or two of the MVCT slices.

**Figure 7:**
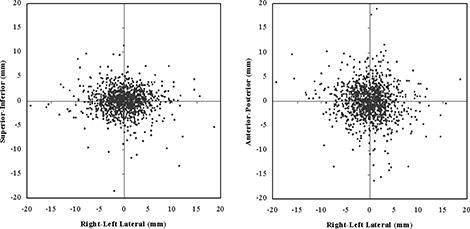
Measured (A) superior‐inferior, (B) anterior‐posterior, and (A,B) lateral patient setup shifts measured from megavoltage computed tomography (MVCT) image fusion for the first 33 prostate patients. Patients were set up based on external laser alignment with skin marks.

For the 33 prostate patients, Fig. 7 shows the total anterior–posterior, superior–inferior, and right–left lateral shifts from the initial patient setup based on a 3‐point alignment of skin marks with wall‐mounted lasers. As expected, the positional error was larger primarily in the anterior–posterior direction because of a combination of external setup error, internal organ motion, and couch sag. The typical range of prostate displacement measured in this study was 2 – 10 mm (3.4 mm SD) in the anterior–posterior direction and 1 – 6 mm (2.4 mm SD) in the superior–inferior direction, which corresponds well with other reported imaging studies.^(^ ^1^ ^–^ ^24^ ^)^ The range of displacements in the lateral direction was 0 – 8 mm (3.7 mm SD).

Table 3 lists the random error (1 SD) for the 30 prostate patients who were imaged 38 – 39 times during the course of therapy. Also listed is an estimate of the geometric misses that would have occurred because of random error without image‐guidance during the course. The percentage of fractions exceeding the treatment margin was calculated from the number of fractions in which the random error would have caused either or both of a greater than 5‐mm shift in the posterior direction and a greater than 10‐mm shift in all other directions.

In 2 patients, we observed substantial lateral shifts (8.1 – 8.4 mm SD), which could have resulted in a lateral geographic miss in more than 20% of the delivered treatment fractions. Both of these patients were morbidly obese, and the laser alignment marks on the skin surface were extremely mobile relative to the prostate.

In 8 patients, large fluctuations in rectal contents occurred, which resulted in an increased anterior–posterior random error (4.0 – 9.4 mm SD) that could have lead to a treatment miss in more than 10% of the delivered fractions. One patient had an extremely distended rectum with a mean diameter of 6.7 cm during the course of treatment. This patient had the potential for a geometric miss in 41% of the treatment fractions.

During this study, deformation in the shape of the prostate or the seminal vesicles because of large changes in rectal and bladder contents was not taken into consideration. The effect that such deformation has on treatment margins requires further investigation. Based on the findings in the present study and the recent clinical data of Crevoisier et al. on the effect of a distended rectum on outcomes, patients with large fluctuations in rectal contents should be treated with larger margins or IGRT, or both, to ensure proper tumor coverage.^(44)^

**Table 3:** The measured random error for each of 30 patients treated and imaged for 38 – 39 fractions; also listed is the percentage of treatment fractions for which the random error would have exceeded the treatment margins without the use of image‐guided radiation therapy^a^

	1 SD Setup error (mm)			Percentage exceeding margin		
Patient	Rt–lt	Sup–inf	Ant–post	Rt–lt	Sup–inf	Ant–post
1	3.8	1.3	2.6	0	0	3
2	3.2	1.8	1.7	0	0	0
3	3.7	1.4	2.1	0	0	0
4	2.7	1.8	3.5	0	0	3
5	2.6	1.6	2.7	0	0	3
6	3.7	4.5	9.4	0	5	41
7	5.2	3.8	2.3	5	0	0
8	3.8	2.7	2.2	0	0	0
9	4.8	1.6	3.8	5	0	8
10	2.7	1.7	1.8	0	0	0
11	3.7	2.0	2.5	0	0	3
12	2.1	1.7	2.1	0	0	5
13	8.4	3.6	5.0	21	0	18
14	3.2	1.3	3.1	0	0	8
15	4.5	1.6	3.1	3	0	3
16	2.6	1.5	2.4	0	0	3
17	1.9	3.0	3.0	0	0	5
18	2.8	2.4	1.8	0	0	0
19	4.1	2.2	2.3	3	0	3
20	3.6	2.7	3.4	3	0	8
21	8.1	5.3	6.7	21	3	13
22	3.9	4.9	2.7	0	5	5
23	3.2	2.0	5.5	0	0	13
24	2.9	3.2	4.2	0	3	8
25	2.3	1.8	4.0	0	0	10
26	3.7	3.2	2.6	0	0	0
27	3.1	2.4	3.4	3	0	11
28	5.0	2.2	5.7	0	0	18
29	3.4	2.7	4.0	0	0	10
30	2.2	1.5	2.0	0	0	0

a The planning treatment volume (PTV) for all patients was defined by adding a 5‐mm margin around the clinical treatment volume (CTV) posteriorly and a 10‐mm margin in all other directions.

SD=standard deviation; Rt−lt=right−left; Sup−inf=superior−inferior; Ant−post=anterior−posterior.

#### C. Treatment delivery

Delivery times for the helical tomotherapy treatments were comparable with those for fixed‐gantry IMRT on a conventional linear accelerators. The mean beam‐on time for tomotherapy prostate patients treated with the 2.5‐cm slice was 302.6 s. In comparison, the delivery time from beam‐on of the first to beam‐off of the fifth multiple‐static‐segment treatment field on a Varian Medical Systems (Palo Alto, CA) linear accelerator was 243.1 s.

The patients in the present study were assessed for early urinary and gastrointestinal (GI) toxicity based on modified RTOG grading criteria.^(43)^ The mean follow‐up time was 24.0 months (range: 14.3 – 38.5 months). Acute tolerance for image‐guided helical tomotherapy was very good. With a mean dose of 76.7 Gy, 23 patients (70%) experienced grade I or no urinary toxicity, and 31 patients (94%) experienced grade I or no rectal toxicity. In addition, no acute grade III or IV toxicities were observed. Although the follow‐up time and the patient cohort are both small, the acute toxicity levels observed in this phase I study are consistent with those reported in other studies. The 6% grade II GI toxicity rate observed in this study is constant with the 14%−16% incidence reported in RTOG 94–06.^(^ ^38^ ^,^ ^45^ ^)^ Likewise, the 30% grade II genitourinary toxicity rate observed in this study is also consistent with the 26% incidence reported in RTOG 94–06.

## IV. CONCLUSIONS

In the present study, we implemented a clinical technique for daily MVCT‐based image‐guided helical tomotherapy. The PTV for all patients was defined by adding a 5‐mm margin around the CTV posterior, and a 10‐mm margin in all other directions. Using MVCT imaging, 33 patients were positioned daily for treatment for a total of 1266 imaging and treatment sessions. The prostate, seminal vesicles, rectum, and bladder anatomy were used to position the patient relative to the desire treatment position without the use of implanted markers. The patients were treated to a mean dose of 76.7 Gy in 2 Gy per fraction. The acute toxicity levels were within the expected range given the number of patients treated and the dose level.

One of the major challenges in prostate radiotherapy is the combination of daily setup error and internal organ motion. With the advent of IGRT and IMRT, the potential now exists to deliver dose escalation to prostate patients that are at moderate‐to‐high risk, without dramatically increasing toxicities. However, increased conformal avoidance and reduced treatment margins increase the risk of a partial geometric or dosimetric miss of the target volume.

Without the use of IGRT, more than 25% of the patients had the potential for a geometric or dosimetric miss of the CTV with posterior margins of 5 mm and margins of 10 mm in all other directions. The two sources of error were the large setup error observed in 2 obese patients and the large rectal organ motion during the course of therapy. Although image‐guidance prevented these errors, further study is needed to determine the minimum IGRT margins that incorporate intrafraction motion and organ deformation.
